# Fractal Characteristics of Corrosion-Induced Cracks in Reinforced Concrete

**DOI:** 10.3390/ma13173715

**Published:** 2020-08-22

**Authors:** Haodong Ji, Haoyu Jiang, Ruoyi Zhao, Ye Tian, Xianyu Jin, Nanguo Jin, Jing Tong

**Affiliations:** 1 Department of Civil Engineering and Architecture, Zhejiang University, Hangzhou 310058, China; jihd@zju.edu.cn (H.J.); haoyujiang@zju.edu.cn (H.J.); jinng@zju.edu.cn (N.J.); 3130104658@zju.edu.cn (J.T.); 2 Department of Art Design, Zhejiang Gongshang University, Hangzhou 310058, China; 21912155@zju.edu.cn

**Keywords:** reinforced concrete structure, corrosion-induced cracks, fractal geometry, characteristic contour parameter, cracking pattern

## Abstract

Based on the fractal geometry, a quantitative index describing the development degree of the internal corrosion expansion of reinforced concrete was proposed. This approach could describe the similarity and complexity of the development of corrosion-induced cracks in concrete simultaneously. Based on this approach, the influence of cracking pattern and coarse aggregate distribution on crack distribution was investigated. This study obtained the crack distribution of reinforced concrete by using the half-soaking galvanic accelerated corrosion method. The results showed that the cracking pattern was the main factor affecting the complexity of crack distribution. For cracks with the simplest cracking pattern, the presence of coarse aggregate and its surface irregularity greatly affected their development trend.

## 1. Introduction

Corrosion cracking is one of the manifestations of durability failure of reinforced concrete structures [1,2]. Among all the reasons causing the corrosion of the steel bar, the macrocell and microcell corrosion induced by chloride ions invasion is relatively common [3,4]. The cross-sectional area of the steel bar will decrease after corrosion, which leads to a decrease in bond property between the steel bar and concrete cover [5]. The volume of corrosion products generated by corrosion is 2–4 times larger than that of the original steel [6]. The continuous expansion of corrosion products will result in the cracking of concrete cover, thus reducing the service life of concrete structures [7,8,9,10,11,12]. Therefore, the accurate assessment of the damage of corroded reinforced concrete structures has become an important and hot issue for the investigation.

Since Griffith et al. [13] established traditional fracture mechanics in 1920, many scholars [14,15,16,17,18,19] have found that brittle materials show notable fractal characteristics. The theory of fractal geometry, founded by French mathematician Mandebrot [14] in the 1970s, is an innovative tool for describing the natural phenomena, which are irregular, complex, and disordered that don’t conform to Euclidean geometry. The method of fractal geometry is applied effectively in many fields of scientific research [20,21], such as biomedical, aerospace, submarine, etc. As the cracking is influenced by so many factors, it is always difficult to characterize the cracking behaviors of materials. The application of fractal geometry in explaining the cracking features of materials was performed almost immediately after it was invented. Fractal applications in cracking problems can be summarized into three categories as the experimental study on fractal characteristics of sections and fractal measurement [14,17], the relationship between fractal dimension and macroscopic mechanical properties [22,23], and the physical mechanism of fractal fracture [24,25,26,27].

In the last decades, scholars mainly focused on the width and development process of cracks on the outer surface of the concrete cover [28,29,30,31,32,33]. Only a few studied the cracking pattern of corrosion-induced expansive cracks in concrete. At present, the fractal geometry is widely [14,15,16,17,18,19] applied to characterize the properties of concrete. However, few of them have studied whether the cracking pattern of cracks has fractal characteristics. In fact, concrete is a kind of multiphase composite material. The stress and strain field inside concrete is very complex, resulting in the formation of intricate cracks. So, it is necessary to determine whether corrosion-induced cracks have obvious fractal characteristics. In addition, the fractal characteristics need to be described by multi-parameter analysis, rather than using a single kind of parameter.

In this paper, corrosion-induced cracks of concrete specimens under different corrosion ratios were characterized using fractal geometry theory for the first time, and a quantitative index that superposes the representation of multi-parameters was proposed to describe the degree of damage induced by corrosion expansion.

## 2. Experimental Investigation

### 2.1. Materials

**Figure 1:**
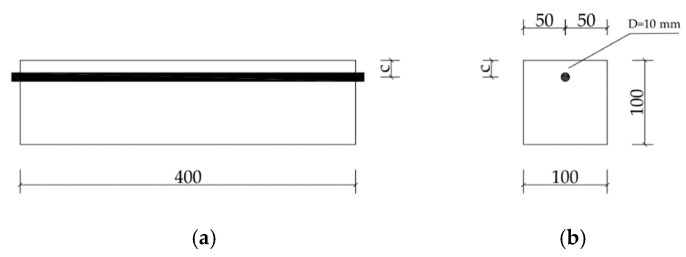
The geometry of the specimen and the positioning of reinforcement: (**a**) Front view; (**b**) Left view. c represents concrete cover thickness.

The mix proportion of concrete is listed in Table 1. The cement used in this test was P·I 52.5 Portland cement produced by the Hubei huaxin cement factory. The mineral composition of cement is shown in Table 2. River sand with the fineness modulus of 2.64 was selected as the fine aggregate. The coarse aggregate was crushed gravel, meeting the continuous grading of 5–20 mm. The water was tap water. All materials were purchased in Hangzhou city of Zhejiang Province in China and no other admixtures were added into the concrete. The size of each specimen was 100 mm × 100 mm × 400 mm, with no stirrups. The specimen was cured under standard curing conditions, and the compressive strength of concrete at 28 d was 46.3 MPa. There was only one reinforcing bar in the specimen. The type of longitudinal bar was hot-rolled plain steel bars (HPB) 235, and the diameter was Φ10, and its chemical composition is shown in Table 3. The size of the specimen and the positioning of reinforcement are shown in Figure 1.

In this test, a total of six specimens were designed, and their parameters are shown in Table 4. In the serial number, L means the corrosion of longitudinal steel bar; 20 or 30 states the thickness of concrete cover; N denotes the no stirrup; the last number specifies the corrosion level.

### 2.2. Experimental Procedure

**Figure 2:**
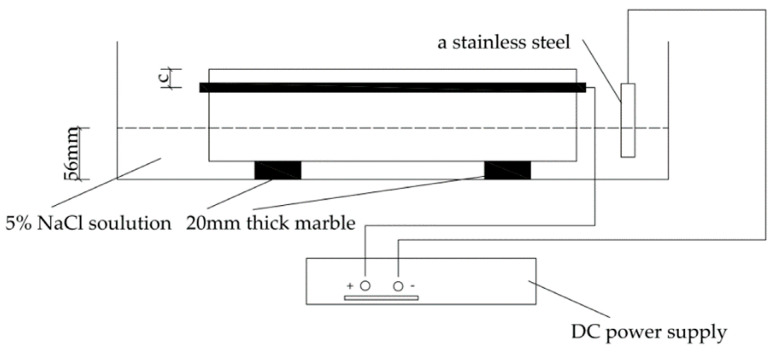
Experimental setup.

Accelerated corrosion can greatly shorten the test period on the premise of achieving the target corrosion effect. In this experiment, a half-soaking galvanic accelerated corrosion method was used to speed up the corrosion process of reinforcing bar. The steel bar was cast into concrete, and only a small part left out of the specimen. A plastic-sheathed copper wire with a diameter of 2.5 mm was welded to it. The exposed part of the steel bar and the copper wire connected to it were coated with epoxy resin against corrosion. Before the experiment, the specimen was totally immersed in a 5% NaCl solution for 72 h. Then, part of the solution was drained out of the tank until part of the specimen was exposed to the air, and the left out remained in the solution. The immersion depth of all specimens was 36 mm. In order to ensure sufficient contact between the specimen and NaCl solution, and sufficient space for vertical deformation of the specimen, two 20 mm thick marble supports were used at the bottom of the specimen. At the same time, stainless steel was put into the solution as an auxiliary electrode (cathode), which was connected with the negative pole of the DC power supply. The steel bar in the specimen acted as the anode, which was connected to the positive pole of the DC power supply. As shown in Table 4, the corrosion time due to the power-on time of different specimens has been listed. During the experiment, the corrosion current was 0.2 A. The liquid level and the concentration of NaCl solution were checked regularly every 12 h to ensure an unchanging submersion condition. The experimental setups of L20N and L30N are shown in Figure 2.

After the accelerated corrosion, corrosion cracks formed and propagated to the surface of specimens. To maintain the original information of the corrosion cracks, the specimens were coated with epoxy resin. Due to the high flowability, the corrosion cracks were completely filled by epoxy resin. After hardening, the shape of corrosion cracks was fixed to a great extent. Then, each specimen was mechanically cut into 16 slices along the longitudinal direction to get more crack information.

## 3. Analysis of Corrosion-Induced Cracking Pattern in Concrete

Corrosion-induced cracks form from the steel-concrete interface. With the continuous accumulation of corrosion products and the increase of corrosion expansion force, cracks gradually propagate to the outer surface of concrete cover. When the cracks penetrate completely through the concrete cover, then the corrosion expansion force is released, and several irreparable cracks are permanently left in concrete cover. Concrete is a kind of multiphase material, in which there exist a certain number of micro-voids and micro-cracks. During the corrosion cracking process, the micro-defects could connect and propagate under corrosion expansion stress. Therefore, the diversity of cracking patterns is mainly caused by the inhomogeneity of concrete material. Previous studies [34,35,36] have shown that the development trend of cracks is mainly affected by the corrosion ratio of steel bar, the thickness of the concrete cover, the particle size of coarse aggregate, and its position in concrete. Among them, the corrosion ratio represents the degree of steel corrosion.

**Figure 3:**
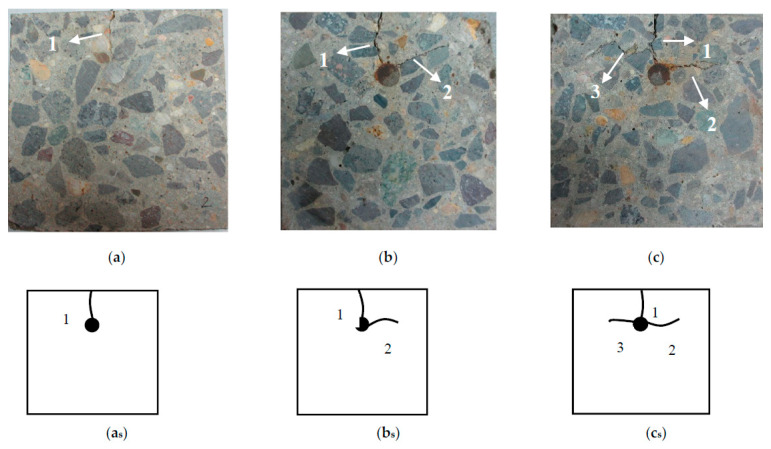
Cracking patterns of corrosion-induced cracks: (**a**) Pattern I; (**b**) Pattern L; (**c**) Pattern T; (**a_s_**) Simplification of the pattern I; (**b_s_**) Simplification of the pattern L; (**c_s_**) Simplification of the pattern T. (Sequence numbers in figures indicate the appearance order of cracks).

By analyzing the cracking patterns of the six specimens, it is found that cracks mainly show three kinds of shapes as pattern I, pattern L, and pattern T. Cracking pattern I extends from the surface of steel bar to thinner concrete cover. As a result, the corrosion crack appears at the top surface of the specimen, which is parallel to the longitudinal steel bar, as shown in Figure 3a. With the increase of corrosion ratio, the corrosion induced by the invasion medium transported from the side surface of the specimen will possibly generate a second crack, which usually appears on the left or right side of the steel bar. In most of the cases, the two cracks form the shape of L, as shown in Figure 3b. It can be considered that the cracking pattern L is the further development of pattern I. While, the micropores, and interfacial transition zones (ITZ) on the surface of coarse aggregates, could influence the cracking path and cracking tendency in concrete cover. Due to the distribution of the corrosion products layer, it can lead to a non-uniform distribution of the stress field; hence, the angle between these two cracks is roughly a right angle [37]. Similarly, with the further increase of corrosion ratio, the third crack may appear in the reverse direction of the second corrosion crack, and the whole cracks show a pattern of inverse “T”, as shown in Figure 3c. In this test, a corrosion crack appears on the right side of the steel bar, so it’s L pattern, and it does not propagate to the surface of the specimen. This phenomenon can be attributed to the corrosion stress release due to the corrosion cracking. The concrete cover on the right side of the steel bar is thicker than the top side. So, the corrosion development will possibly cause an increase of width for the corrosion crack on the top side of the specimen rather than the cracking propagation on the right or left side. So, under the corrosion ratio achieved in this research, the corrosion cracks in L and T patterns don’t all reach the surface of the specimen.

The experiment results imply that the real situation of corrosion-induced cracks cannot be fully reflected only by observing cracks on the surfaces of the specimen. Some cracks do not reach the specimen surface, but they have caused severe damage to the concrete specimen. The cracks observed on the surface are parallel to the longitudinal bar, but the undiscovered corrosion cracks may have appeared around the longitudinal bar.

To further explore these cracks, each specimen was cut into 16 slices under the protection of epoxy resin. Since the cut surface between two slices is used as the measuring point, each specimen has 17 measuring points. The concrete cover of each slice was broken mechanically, and the corroded steel bar was obtained to measure the actual corrosion ratio. Due to the loss of steel bar length caused by mechanically cutting, the actual length of each piece of steel bar was measured by Vernier caliper. The actual corrosion ratio *η_i_* of each slice can be obtained as follows.

(1) $$\eta_{i}=\frac{\mu l_{i}-m_{i}}{\mu l_{i}}\times 100\%$$

In Equation (1), *µ* is the mean mass of uncorroded steel bar per unit length, that is, 0.6167 g/mm; *l_i_* is the actual measured length of an *i*th piece of steel bar; *m_i_* is the actual mass of an *i*th piece of steel bar after corrosion. The corrosion ratio of the specimen, *η*, is represented as the mean value of the 16 slices, which is calculated as:

(2) $$\eta =\frac{\sum_{i=1}^{16}\eta_{i}}{16}$$

The type of cracking pattern in each cut surface was recorded. The corrosion ratios and the number of cracking patterns for all specimens are shown in Table 5.

According to Table 5, the actual corrosion ratios of L20N-1 and L30N-1 are both less than 3%. Due to the low corrosion ratio, cracking patterns of L20N-1 and L30N-1 are both patterns I, that is, only one crack extends from the surface of the steel bar to concrete cover. The corrosion ratios of L20N-2 and L30N-2 are about 7%. The cracking patterns contain both pattern I and pattern L. Most of the cracking patterns of them are patterns L. This suggests that with the increase of corrosion ratio, the second crack appears near the steel bar. Finally, with the progress of corrosion, cracking pattern T appears in the specimen, and the number exceeds pattern L remarkably, which can be seen in L20N-3 and L30N-3.

By comparing the cracking patterns of sections of L20N-2 and L30N-2, it can be found that under the similar corrosion ratio, the number of cracks L in L30N-2 is smaller than that in L20N-2. The explanation for this is, the larger the thickness of the concrete cover, the greater the energy required for cracks to reach the cover surface. A practical conclusion of the explanation is that when the corrosion ratio is relatively unchanged, increasing concrete cover thickness can reduce the probability of multiple cracks in concrete and prevent cracks from reaching the surface.

## 4. Description of Fractal Characteristics of Corrosion-Induced Cracks

### 4.1. Crack Distribution Described by Fractal Geometry

At present, some scholars apply fractal geometry to the study of the fracture section of metal materials [38] and the research of brittle materials, such as ceramics [39,40] and geotechnical materials [41]. However, not all irregular shapes have fractal characteristics. Only those that satisfy self-similarity within a certain scale have fractal characteristics. Concrete is a kind of composite material. Its stress is very complex, resulting in the formation mechanism of intricate corrosion-induced cracks. First of all, it is necessary to determine whether corrosion-induced cracks have fractal characteristics.

**Figure 4:**
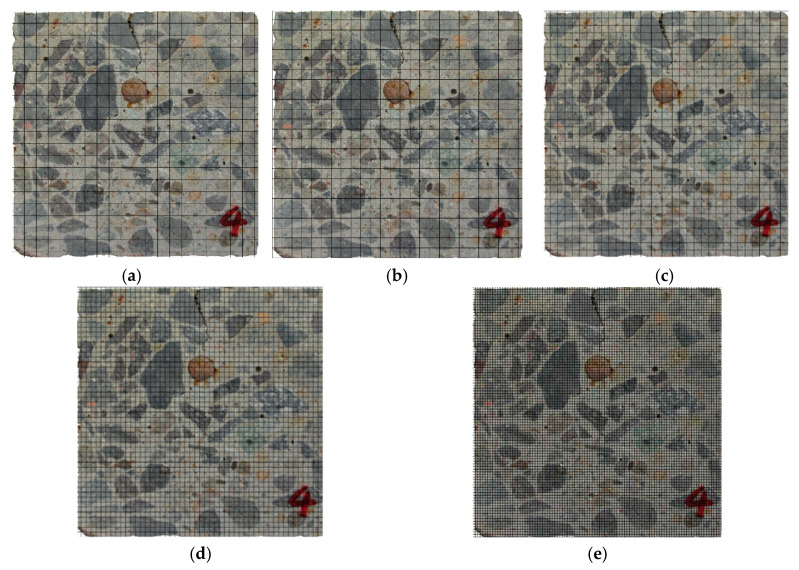
Meshing of digital pictures with different scale distances: (**a**) r = 5 mm; (**b**) r = 4 mm; (**c**) r = 3 mm; (**d**) r = 2 mm; (**e**) r = 1 mm.

The cut surfaces of the slices were photographed by BASLER A406K digital camera. Then, the digital photographs were meshed on a computer using Photoshop with five kinds of grid-scale. As shown in Figure 4, the side length of the grids, *r*, was 1, 2, 3, 4, and 5 mm, respectively.

According to Xu et al. [38], if the cracks have fractal characteristics, there is a relationship between *N* and *r*. The number *N* of the grids occupied by cracks was counted. *N* was defined as the minimum of the grids required to cover the cracks, that is, any grid that contained the cracks belonged to *N*. The number *r* is the side length of the grids.

(3) $$N=C\cdot r^{-D}$$

where *C* is the scale coefficient, and *D* is the fractal dimension. A linear relationship can more directly reflect this relationship in the figure. So, Equation (3) takes lg of both sides. lg*N* and lg(1/*r*) represent the intercepts of the linear relationship on the Y-axis and X-axis, respectively. Each specimen was mechanically cut into 16 slices along the longitudinal direction. By counting the grids occupied by cracks shown in Figure 3, the relationship between lg*N* and lg(1/*r*) can be obtained. If lg*N* is linearly related to lg(1/*r)*, it can be concluded that the cracks satisfy self-similarity, and crack distribution can be described by fractal theory. According to the definition of box dimension given by Mandelbrot [14], the slope of the relation curve represents fractal dimension *D*. The vertical intercept of the relation curve is lg(*C*). Therefore, the relationship can be obtained as follows.

(4) lgN=D·lg(1/r)+lg(C)

Previous studies [42,43,44] have shown that fractal dimension *D* reflects the irregularity of the profile curve with fractal characteristics. It belongs to the parameters used to measure the similarity of the surface profile. The change of *D* reflects how exactly the profile curve satisfies the similarity. Meanwhile, the scale coefficient *C* reflects the size of the surface profile at the unit scale. It belongs to the parameters of absolute counting of surface profile, that is, the variation of roughness. When the above parameters are used to describe crack distribution in concrete, *D* represents the similarity of crack distribution, and *C* represents the complexity of crack distribution. The more cracks there are, the longer and wider they will be, and the more complex the crack distribution will be.

### 4.2. Fractal Characteristics of Corrosion-Induced Cracks in Specimens with Different Corrosion Ratios

#### 4.2.1. Analysis of Fractal Characteristics of the Specimens

**Figure 5:**
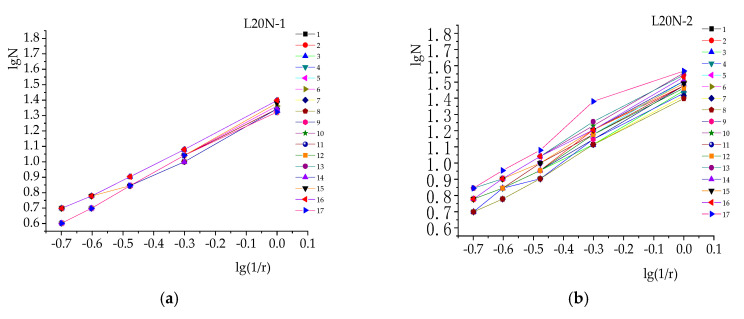

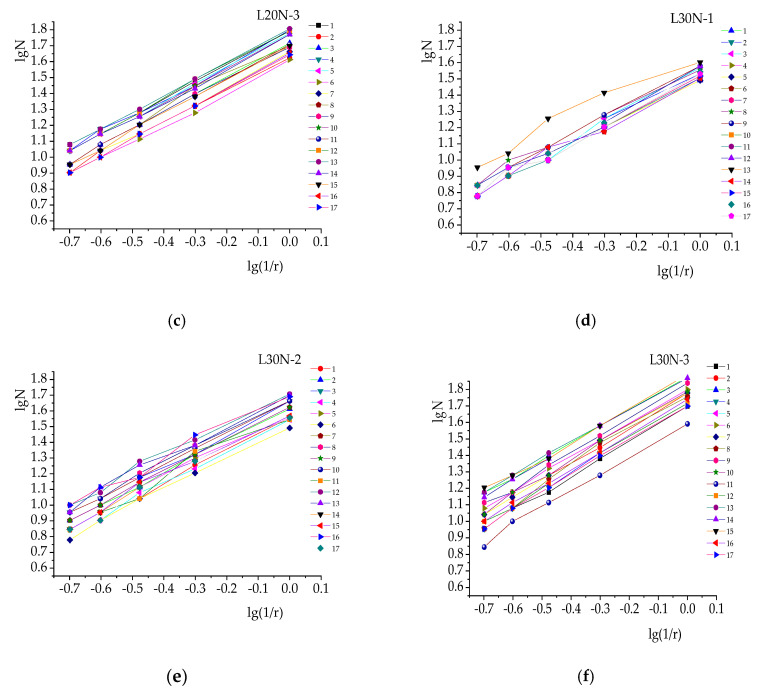
Analysis of fractal characteristics (relationship between lg(1/*r*) and lg*N*) of the different specimens: (**a**) L20N-1; (**b**) L20N-2; (**c**) L20N-3; (**d**) L30N-1; (**e**) L30N-2; (**f**) L30N-3.

Each specimen was cut into 16 slices. The calculation results of lg(1/*r*) and lg*N* for six specimens were substituted into Equation (4) for regression statistics. Figure 5 shows the analysis of the relationship between lg(1/*r*) and lg*N*.

As can be seen from Figure 5, there is an average linear relationship between lg(1/*r*) and lg*N* in each specimen. For several measurement points, such as point 13 of L30N-1, the linear relationship is obvious when *r* is large, while it is not obvious when *r* is small. The reason for this is that small *r* means a dense number of grids. As the subject is not strictly a self-similar figure, the fractal dimension *D* could not be calculated by mathematics. Thus, there are some subjective biases in calculating the number of grids occupied by crack edges by using box-counting. All the correlation coefficients are above 0.95, and so it could be considered that there is a good linear relationship here. Therefore, it can be concluded that the cracks in concrete have obvious fractal characteristics. It is reasonable to describe them by using fractal geometry theory.

#### 4.2.2. Fractal Dimension D and Scale Coefficient C

**Figure 6:**
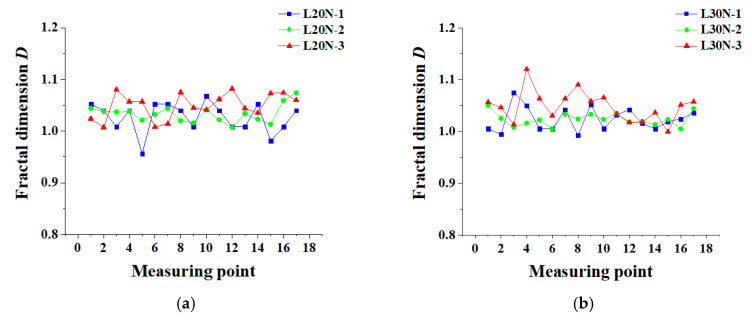
Variation of fractal dimension *D*: (**a**) L20N; (**b**) L30N.

Variation of the fractal dimension *D* of each specimen along the longitudinal axis is shown in Figure 6. Detailed fractal dimensions *D* in this test are shown in Table A1.

**Figure 7:**
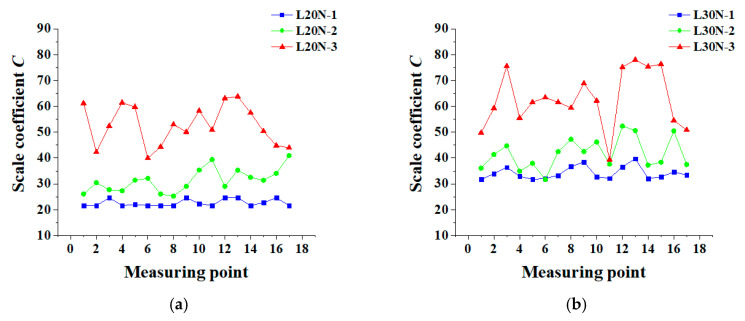
Variation of scale coefficient *C*: (**a**) L20N; (**b**) L30N.

As can be seen from Figure 6, most of the fractal dimensions are between 1.0 and 1.1. According to Li et al. [45], the fractal dimension mainly describes the complexity of cracks, which can reflect the propagation of cracks. From microcracks to a whole crack, and to multiple cracks, the complexity of cracks increases, so does the fractal dimension *D* from around 1.0 to 1.3. In this study, the value of *D* ranging from 1.0 to 1.1 can be attributed to that only a few cracks appear in the experiment. It is believed that *D* in Figure 6 is nearly unchanged. Although the corrosion ratio is aggravated, *D* does not change clearly. This furtherly shows that the corrosion-induced cracks have fractal characteristics. In Figure 6a, the fractal dimension *D* for 65% measuring points of L20N-3 is higher than that of L20N-1. In specimens L30N, there are 14 measuring points, where the relation of *D* shows L30N-3 > L30N-1, accounting for 82%. This phenomenon indicates that as the corrosion time increases, the cracks gradually develop from a pattern I to pattern T, and the shapes of the cracks become more irregular. However, simply using *D* to describe the cracks is not sufficient and objective. In order to describe the crack distribution more accurately, the scale coefficient *C* should also be taken into account. The longitudinal distribution of *C* is shown in Figure 7. Detailed scale coefficients *C,* in this test, are listed in Table A2.

As can be seen from Figure 7, with the increase of corrosion rate, the corrosion ratio increases sharply. Development of cracking patterns gradually evolves from a pattern I to pattern T. There are more and more cracks, and the width of cracks gets higher and higher. As a result, the scale coefficient *C* increases greatly, more obviously than *D*. Although the fractal dimension *D* can describe the complexity of cracks to a certain extent, it mainly describes whether the cracks satisfy self-similarity. In different slices of the specimen, the scale coefficients vary greatly. This is mainly because the cracking patterns in different slices are not the same, and coarse aggregate distribution also plays an important role. The effect of cracking pattern T on the complexity of cracks is greater than that of pattern L, and the pattern I, in turn. L20N-2, L20N-3, L30N-2, and L30N-3 all have two cracking patterns. Therefore, their scale coefficients change greatly. Soon, the multiple cracking patterns on the same beam will be studied, and it will possibly help to study the nature of cracking. For L20N-1 and L30N-1, there is the only cracking pattern I in them, so the fluctuation of *C* is relatively small. In this case, the main factor affecting *C* is coarse aggregate distribution. In the opinion of Carpinteri [46] and Carpinteri et al. [47], the fractal characteristic is related to complexity and the size of the microstructure in the material. For heterogeneous concrete, the complexity mainly refers to the influence of coarse aggregate distribution on fractal characteristics.

#### 4.2.3. Analysis of Characteristic Contour Parameter

Xu et al. [38] studied the relationship between surface roughness of steel bar and fractal dimension and scale coefficient relatively. Xu et al. found that rebar surface roughness could not be reflected only by *D* or *C*. Therefore, Xu et al. proposed characteristic contour parameter r*, which superposed the representation of *D* and *C* on surface roughness. The calculated r* is more sensitive to the change of surface roughness.

(5) $$r^{*}=C^{\frac{1}{D}}$$

**Figure 8:**
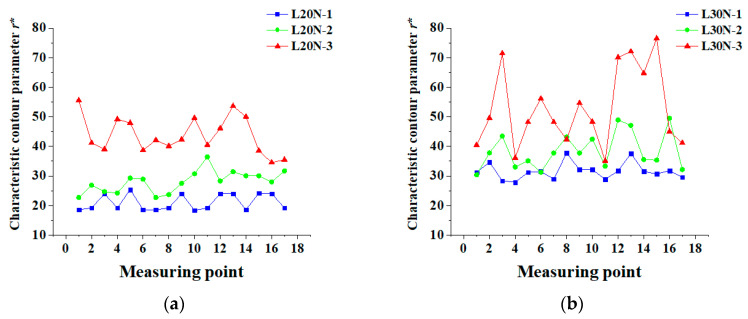
Distribution of r* along the longitudinal axis: (**a**) L20N; (**b**) L30N.

This study drew on the research of Xu et al. and used r* to describe the crack distribution in concrete. It found that the larger the value of r*, the greater the complexity of crack distribution. The characteristic contour parameters r* along the longitudinal axis are shown in Figure 8. Detailed characteristic contour parameters r*, in this test, are shown in Table A3.

As shown in Figure 8, the development of r* with corrosion ratio is similar to that of *C*. The complexity of crack distribution is mainly affected by coarse aggregate and cracking patterns. For L20N-1 and L30N-1, their cracking patterns are the simplest. So, the complexity of crack distribution is mainly affected by coarse aggregate distribution. The maximum rates of change of r* in different sections of the same specimen are 38.1% and 35.3%, respectively. Even though the coarse aggregate distribution in the two specimens is different, the change of r* is not so obvious. However, for L20N-3 and L30N-3, they do not contain cracks I. Their cracking patterns are various. Complexity is both influenced by the coarse aggregate distribution and cracking pattern. The maximum rates of change of r* are 61% and 118%, respectively. L30N-3 has a much larger change in r* than L20N-3. It can be seen that the distribution of coarse aggregate will not cause a complicated crack distribution. The cracking pattern has more influence on the complexity of crack distribution than the coarse aggregate. With the increase of the corrosion ratio, the number of cracks in concrete increases. The cracking pattern of corrosion-induced cracks gradually evolves from a pattern I to pattern T. Characteristic contour parameters r* of the specimens obviously show a rising trend with the growth of corrosion ratio. There are two cracking patterns in L20N-2, L20N-3, L30N-2, and L30N-3. Therefore, in different slices of a specimen, the characteristic contour parameters r* vary greatly along the longitudinal axis. Meanwhile, only one cracking pattern exists in L20N-1 and L30N-1. The main factor influencing r* is the coarse aggregate distribution. By comparing the curves of L20N-1 and L30N-1 in Figure 7 and Figure 8, in different slices of a specimen, the change of r* along the longitudinal axis is more obvious than that of *C*. This conclusion shows that it is more suitable to use characteristic contour parameter to reflect the complexity of corrosion-induced cracks. Mean values of *C*, *D,* and r* are shown in Table 6.

It can be seen from Table 6 that when concrete cover thickness remains unchanged, the larger the corrosion ratio is, the more complex the development of corrosion-induced cracks is. Meanwhile, the increase in the mean value of *C* will be larger. In contrast, the mean value of *D* will change slightly. Because r* superposes the effect of *D* and *C* to describe the complexity of crack development more authentically, with the increase of corrosion ratio, the mean r* increases.

## 5. Analysis of Distribution and Types of Corrosion-Induced Cracks

In practical engineering applications, when the corrosion expansion force reaches the ultimate tensile strength of concrete, it is considered that the inner surface of concrete cover begins to crack. At first, the crack width is small, and the cracking has little influence on the overall strength and stability of concrete. However, once cracks reach the surface of the concrete cover, they provide access for harmful substances from outside to penetrate the concrete cover. Therefore, exploring the complexity of cracks in the initial stage of cracking can provide a basis for researching the transfer rate of harmful substances. In this research, the distribution area of corrosion cracks and the complexity of cracking pattern I are studied. Pattern L and T can be thought of as being made up of many cracks I, and their characteristics are based on cracking pattern I.

### 5.1. Analysis of Distribution Area of Corrosion-Induced Cracks

**Figure 9:**
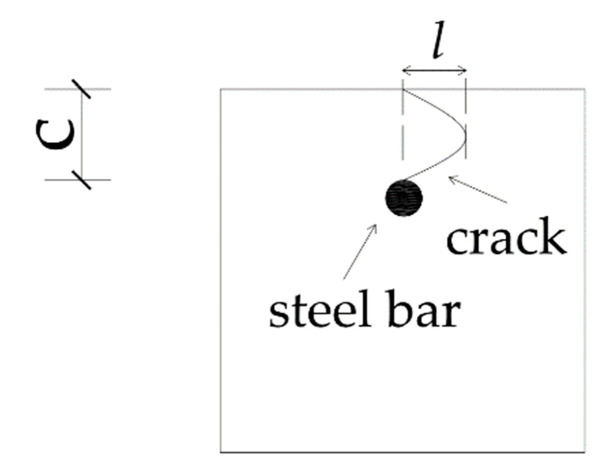
Cracking pattern I.

Although the random distribution of coarse aggregate causes varied tortuosity of cracks, the distribution of cracks is always within a certain range, which is the area directly above the steel bar. Figure 9 shows a simplified crack development. As shown in the figure, *l* represents the spacing between the leftmost and rightmost end of crack development. Table 7 shows the mean value of *l* for cracking pattern I.

In this test, l ranges from 0.73 to 0.92 times of concrete cover thickness. The mean value of all the cracked slices for all the specimens is 0.82 C. Therefore, it can be assumed that the distribution range of cracks in concrete is approximately 80% to concrete cover thickness. Since the concrete cover thickness is known, the general range of crack development can be roughly estimated. In practice, by focusing the object region above the steel bar, the area of concrete members that may propagate corrosion-induced cracks can be monitored and maintained.

### 5.2. Analysis of the Patterns of Corrosion-Induced Cracks

Under the same corrosion ratio, the complexity of corrosion cracks mainly depends on the distribution of coarse aggregate for cracking pattern I. The coarse aggregate will influence the complexity of corrosion cracks from two aspects. The coarse aggregate firstly affects the transfer of an external harmful substance, which leads to the non-uniform corrosion of the steel bar. Secondly, the random distribution of coarse aggregate will increase the tortuosity of cracking propagation. In comparison with cement mortar, concrete is prone to cracking along the interfacial zone (ITZ) on the surface of coarse aggregate. A random distribution of coarse aggregate can induce tortuous cracks. Therefore, it is necessary to furtherly clarify the influence of coarse aggregate on the formation of the corrosion-induced cracking pattern I.

**Figure 10:**
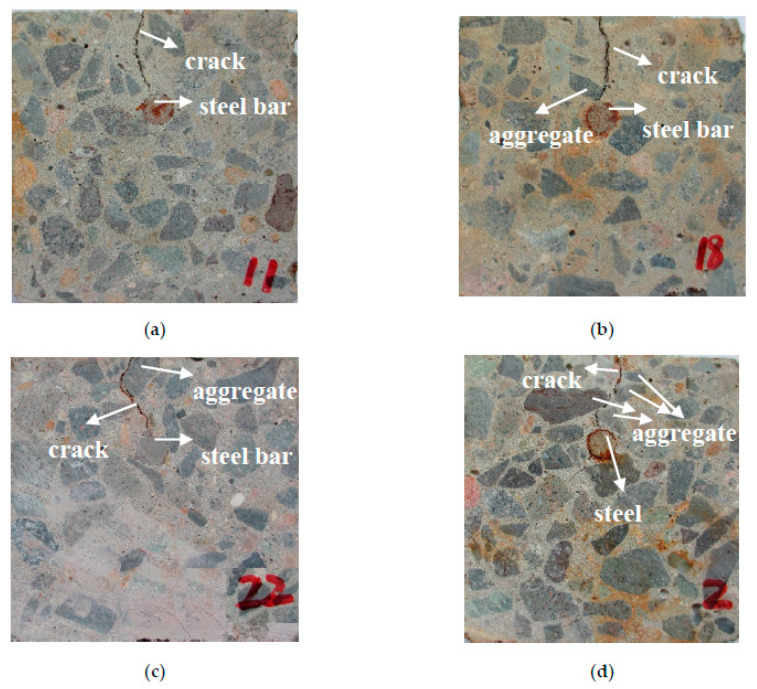
Specified cracking patterns of pattern I: (**a**) Cracking pattern I-1; (**b**) Cracking pattern I-2-1; (**c**) Cracking pattern I-2-2; (**d**) Cracking pattern I-2-3.

According to whether the cracks have contact with coarse aggregate or not, the cracks in cracking pattern I are divided into cracks I-1 that have no contact with coarse aggregate and cracks I-2 that have contact. Furtherly, according to the contact position of coarse aggregate, cracks I-2 are furtherly specified as cracks I-2-1 that are in contact with aggregates deep in the concrete cover, cracks I-2-2 that are in contact with aggregates near the surface of concrete cover, and cracks I-2-3 that are in contact with both internal and surficial aggregates. The detailed cracking patterns of these cracks are shown in Figure 10.

As can be seen from Figure 10, compared with the other three types of cracks, the direction of crack I-1 development is single, and the crack width is smaller. Since cracks I-2-1, I-2-2, and I-2-3 are in contact with aggregates in different positions, their tortuosity is larger than cracks I-1, and they develop along the edge of the aggregates. Because cracks cannot penetrate the aggregates, the complexity of their trajectories depends on how irregular the shape of the aggregates is. The more irregular the shape of aggregate is, the greater the tortuosity of the crack is, and the more difficult it is for harmful substances to penetrate deep into the concrete cover. Because the cracks do not develop along the entire edge of the aggregates, it is not represented to study the shape and size of a single aggregate. In other words, the edge of the aggregates only partly affects the cracking patterns. Cracks I-2-1 are in contact with aggregates deep in the concrete cover, so their widths are larger than cracks I-1. Thus, the presence of aggregate will increase the degree of cracking. Due to the low strength of interface transition zone (ITZ), concrete cover cracks along ITZ under the corrosion expansion force. For cracks I-2-2 in contact with aggregates near the concrete cover surface, they develop a high tortuosity, and their widths near the surface are smaller than that close to the steel bar. This suggests that the cracking rate accelerates significantly when the crack reaches ITZ, which further proves that the aggregate makes the cracking worse. For cracks I-2-3 in contact with both internal and surficial aggregates, their widths are larger than cracks I-2-2, and their development patterns are more complex. In summary, the development complexity of cracks I-2-3 are greater than cracks I-2-2, and that of cracks I-2-2 are greater than cracks I-2-1. The development patterns of cracks I-1 are the simplest of the four types. For cracks with a single development trend, the presence of coarse aggregate and its surface irregularity greatly affect the development of cracks.

## 6. Conclusions

The development pattern of corrosion-induced cracks in reinforced concrete has fractal characteristics and obvious self-similarity, which can be described by fractal geometry. In this paper, the fractal dimension *D*, scale coefficient *C,* and characteristic contour parameter r* are proposed to describe the characteristics of cracks.

- The self-similarity of the cracking pattern can be described by fractal dimension *D*, the value is mainly between 1.0 and 1.1, and there is a slight increase with the corrosion rate. The complexity of the cracking pattern can be described by scale coefficient *C*, and the value is mainly between 20 and 60. The greater the complexity of the crack, the greater the value of *C*. Based on *D* and *C*, characteristic contour parameter r* is proposed, and it’s more accurate to describe the distribution of cracks. The larger the r*, the more complex the crack distribution.
- For cracks in general, the influence of cracking patterns on the complexity of crack distribution is greater than the coarse aggregate. With the corrosion ratio increasing, the number of cracks increases, and the cracking pattern gradually evolves from the pattern ‘I’ to pattern ‘T’. Meanwhile, the characteristic contour parameter r* increases. Therefore, the cracking pattern is the main factor affecting the complexity of crack distribution in concrete.
- For cracks with a single development trend, the presence of coarse aggregate and its surface irregularity greatly affect the development of cracks. The presence of aggregate will increase the degree of cracking. The more irregular the aggregate surface is, the greater the tortuosity of the crack is, and the more difficult it is for harmful substances to penetrate deep into concrete cover.

## Figures and Tables

**Table 1:** Mix proportions of concrete.

Materials	Cement	Water	Fine Aggregate	Coarse Aggregate
Mass ratio	1	0.53	2	3

**Table 2:** The mineral composition of P·I 52.5 Portland cement (mass %).

Ingredient	*C*_3_ *S*	*C*_2_ *S*	*C*_3_ *A*	*C*_4_ *AF*	Gypsum
Proportion	55.5	19.1	6.5	10.1	5

***C*_3_*S*** :3*CaO*·*SiO*_2_, ***C*_2_*S*** :2*CaO*·*SiO*_2_, ***C*_3_*A*** :3*CaO*·*Al*_2_O_3_, ***C*_4_*AF*** : 4*CaO*·*Al*_2_*O*_3_·*Fe*_2_*O*_3_

**Table 3:** The chemical composition of HPB235 reinforcement (mass %).

Element	*C*	*Mn*	*Si*
Proportion	0.22	0.65	0.30

HPB235:hot-rolled plain steel bars 235.

**Table 4:** Parameters of the specimens.

Number	Concrete Cover Thickness (mm)	Corrosion Time (h)
L20N-1	20	84
L20N-2	20	168
L20N-3	20	336
L30N-1	30	84
L30N-2	30	168
L30N-3	30	336

**Table 5:** The number of cracking patterns in each specimen.

Specimen	Corrosion Ratio *η* (%)	Pattern I	Pattern L	Pattern T
L20N-1	2.72	17	0	0
L20N-2	6.91	4	13	0
L20N-3	11.20	0	4	13
L30N-1	2.98	17	0	0
L30N-2	6.78	7	10	0
L30N-3	10.70	0	6	11

**Table 6:** Mean values of the parameters.

Number	Mean Values of *D*	Mean Values of *C*	Mean Values of r*
L20N-1	1.027	22.62	21.06
L20N-2	1.033	31.37	28.06
L20N-3	1.049	52.75	43.79
L30N-1	1.023	34.19	31.61
L30N-2	1.022	41.72	38.86
L30N-3	1.048	62.73	52.93

**Table 7:** The distribution range of corrosion-induced cracks.

Number	*l*^1^ of Pattern I	$\mathrm{Mean}\;\mathrm{Value}\;\bar{l}$
L20N-1	0.80 C ^2^	0.82 C
L20N-2	0.92 C	
L30N-1	0.83 C	
L30N-2	0.73 C	

^1^*l* represent represents the spacing between the leftmost and rightmost end of crack development, ^2^ C represents concrete cover thickness.

## Appendix A

**Table A1:** The fractal dimension *D.*

Measuring Point	L20N-1	L20N-2	L20N-3	L30N-1	L30N-2	L30N-3
1	1.052	1.044	1.024	1.005	1.050	1.056
2	1.040	1.038	1.007	0.994	1.025	1.046
3	1.008	1.037	1.080	1.075	1.008	1.013
4	1.040	1.038	1.057	1.050	1.016	1.120
5	0.956	1.021	1.057	1.005	1.022	1.063
6	1.052	1.032	1.008	1.005	1.005	1.030
7	1.052	1.044	1.014	1.042	1.033	1.063
8	1.040	1.020	1.075	0.992	1.024	1.090
9	1.008	1.016	1.045	1.052	1.033	1.058
10	1.068	1.041	1.041	1.005	1.023	1.065
11	1.040	1.022	1.062	1.032	1.035	1.033
12	1.008	1.007	1.082	1.042	1.018	1.017
13	1.008	1.034	1.044	1.015	1.019	1.018
14	1.052	1.023	1.035	1.005	1.013	1.036
15	0.981	1.013	1.073	1.019	1.023	0.999
16	1.008	1.059	1.074	1.024	1.005	1.051
17	1.040	1.074	1.060	1.036	1.044	1.057

**Table A2:** The scale coefficient *C.*

Measuring Point	L20N-1	L20N-2	L20N-3	L30N-1	L30N-2	L30N-3
1	21.64	26.06	61.16	31.72	36.05	49.75
2	21.60	30.44	42.28	33.91	41.35	59.29
3	24.58	27.73	52.36	36.41	44.71	75.51
4	21.60	27.31	61.40	32.91	34.91	55.49
5	22.00	31.41	59.73	31.72	37.93	61.56
6	21.64	32.14	39.90	32.09	31.72	63.37
7	21.64	26.06	44.35	33.27	42.49	61.56
8	21.60	25.23	52.95	36.68	47.24	59.40
9	24.58	29.02	50.07	38.46	42.49	68.84
10	22.39	35.33	58.24	32.72	46.18	62.10
11	21.60	39.38	50.89	32.14	37.66	39.37
12	24.58	28.98	63.10	36.59	52.38	75.16
13	24.58	35.28	63.78	39.72	50.59	77.95
14	21.64	32.50	57.48	32.08	37.24	75.35
15	22.73	31.37	50.36	32.70	38.35	76.31
16	24.58	34.05	44.81	34.57	50.47	54.55
17	21.60	40.93	43.95	33.45	37.47	50.86

**Table A3:** The characteristic contour parameter *r*.*

Measuring Point	L20N-1	L20N-2	L20N-3	L30N-1	L30N-2	L30N-3
1	18.58	22.71	55.56	31.23	30.37	40.46
2	19.18	26.85	41.14	34.66	37.79	49.55
3	23.98	24.65	39.03	28.35	43.43	71.43
4	19.18	24.23	49.15	27.88	33.01	36.11
5	25.39	29.25	47.91	31.23	35.12	48.18
6	18.58	28.90	38.70	31.52	31.23	56.16
7	18.58	22.71	42.13	28.93	37.75	48.18
8	19.18	23.69	40.09	37.73	43.17	42.35
9	23.98	27.50	42.32	32.08	37.74	54.63
10	18.38	30.72	49.57	32.11	42.43	48.32
11	19.18	36.43	40.46	28.90	33.30	35.05
12	23.98	28.27	46.09	31.68	48.94	70.05
13	23.98	31.43	53.62	37.58	47.08	72.05
14	18.58	30.02	50.04	31.51	35.52	64.74
15	24.16	30.03	38.53	30.68	35.35	76.50
16	23.98	27.96	34.53	31.80	49.49	44.98
17	19.18	31.69	35.48	29.57	32.12	41.16
